# Sequence Characterization of *DSG3* Gene to Know Its Role in High-Altitude Hypoxia Adaptation in the Chinese Cashmere Goat

**DOI:** 10.3389/fgene.2018.00553

**Published:** 2018-11-19

**Authors:** Chandar Kumar, Shen Song, Lin Jiang, Xiaohong He, Qianjun Zhao, Yabin Pu, Kanwar Kumar Malhi, Asghar Ali Kamboh, Yuehui Ma

**Affiliations:** ^1^The Key Laboratory for Farm Animal Genetic Resources and Utilization of Ministry of Agriculture of China, Institute of Animal Science, Chinese Academy of Agricultural Sciences, Beijing, China; ^2^Department of Animal Breeding and Genetics, Faculty of Animal Husbandry and Veterinary Sciences, Sindh Agriculture University, Tando Jam, Pakistan; ^3^Department of Veterinary Microbiology, Faculty of Animal Husbandry and Veterinary Science, Sindh Agriculture University, Tando Jam, Pakistan

**Keywords:** *DSG3*, exons, SNPs, Tibetan goat, hypoxia, high-altitude adaptation

## Abstract

The Tibetan cashmere goat is one of the main goat breeds used by people living in the plateau. It exhibits the distinct phenotypic characteristics observed in lowland goats, allowing them to adapt to the challenging conditions at high altitudes. It provides an ideal model for understanding the genetic mechanisms underlying high-altitude adaptation and hypoxia-related diseases. Our previous exome sequencing of five Chinese cashmere breeds revealed a candidate gene, *DSG3* (Desmoglein *3*), responsible for the high-altitude adaptation of the Tibetan goat. However, the whole *DSG3* gene (44 kbp) consisting of 16 exons in the goat genome was not entirely covered by the exome sequencing. In this study, we resequenced all the 16 exons of the DSG3 gene in ten Chinese native goat populations. Twenty-seven SNP variants were found between the lowland and highland goat populations. The genetic distance (*F_ST_*) of significant SNPs between the lowland and highland populations ranged from 0.42 to 0.58. By using correlation coefficient analysis, linkage disequilibrium, and haplotype network construction, we found three non-synonymous SNPs (R597E, T595I, and G572S) in exon 5 and two synonymous SNPs in exons 8 and 16 in *DSG3.* These mutations significantly segregated high- and low-altitude goats in two clusters, indicating the contribution of *DSG3* to the high-altitude hypoxia adaptation in the Tibetan goat.

## Introduction

Low oxygen or hypoxia is the hardest environmental challenge for humans and animals existing at high altitude. Whole-genome resequencing analyses have been carried out to investigate the genetic basis of the hypoxia adaptation in many domestic animals, such as yak (Qiu et al., 2012), Tibetan pig (Ai et al., 2014), dog (Li et al., 2014), and birds (Cai et al., 2013). Various candidate genes have been identified for high-altitude adaptation, including *EPAS1* (endothelial PAS domain protein 1) and *HBB* (hemoglobin beta) (Gou et al., 2014; Fan et al., 2015; Song et al., 2016). In prior studies, both *EPAS1* and *HBB* revealed six non-synonymous mutations potentially affecting the gene function and influencing high-altitude hypoxic adaptation in dogs (Fan et al., 2015). These studies proved that the domestic animals are a useful animal model to explore high-altitude adaptation.

The domestic goat is found at sea level (30 m) to the high plateau (4700 m). In contrast to its low-altitude counterpart, the Tibetan goat has unique anatomical and physiological characteristics such as higher hemoglobin concentration, a larger heart, and bigger lungs that equip it to live at high altitudes (Li et al., 2004b). Our previous exome-sequencing analysis also reveals that the cardio-vascular system-related genes may play a crucial role in high-altitude adaptation (Song et al., 2016). Among the candidate genes under strong selection, the *DSG3* (Desomoglein 3) loci was the most significant. *DSG3* is a desmoglein gene located in a cluster on goat chromosome 24 containing 44 kbp that encodes 16 exons. It is expressed in stratifying epithelia, but its precise role is not fully understood in the cardiovascular system (Delva et al., 2009). Here, we extended our previous analyses on large goat populations from different altitudes to identify if the *DSG3* candidate gene has a role in high-altitude hypoxia adaptation. By using correlation coefficient analysis, linkage disequilibrium, and haplotype network construction, we explored both the non-synonymous and synonymous mutation sites of the *DSG3* gene. These mutations significantly segregated goat populations between high- and low-altitude groups.

## Materials and Methods

### Populations Sampling

A total of 125 Chinese cashmere goats were used for Sanger sequencing and covered the entire genomic region of *DSG3* (44 kbp) consisting of 16 exons. Isolated genomic DNA used from Ritu (RT, 4700 m), Bange (BG, 400 m), Nanjiang (NJ, 1700 m), Inner Magnolia (IM, 1500 m), and Liaoning (LN, 30 m) cashmere goats selected from 330 individuals was analyzed previously for Exome Sequencing by the Illumina Hiseq2000 Platform (Song et al., 2016), which were randomly selected from 4 different locations (Table 1 and Supplementary Table S1 and Supplementary Figure S1). Furthermore, five goat populations including Dulan (DL, 3000 m), Hanshan (HS, 1000 m), Guangfeng (GF, 500 m), Hainan (HN, 120 m), and Changjiangsanjiaozhou (CZ, 50 m) were selected from five different geographical locations in China (Table 1 and Supplementary Table S1 and Supplementary Figure S1). The tissue samples of these goat populations were taken by cutting ear tissue, using an ear-cutting instrument without applying an anesthetic or analgesic agent. After the collection, the tissue samples were preserved in a tube containing 75% alcohol, which was kept in the ice box during transportation to the laboratory. The tissue samples were collected from all the animals undergoing the experiment according to the recommendations and guideline of the Institute of Animal Sciences (IAS, CAAS), with full approval from the Animal Care and Use Committee of Chinese Academy of Agricultural Sciences and the Ministry of Agriculture of the People’s Republic of China.

**Table 1:** Sample locations and major allele frequency of three non-synonymous variants and two synonymous mutations in DSG3 gene.

Sample group	Sample location	Altitude (m)	SIZE	SNP1 (R597E)	SNP2 (T595I)	SNP3 (G572S)	SNP4	SNP5
LN	Gai Zhou, Liaoning	30 m	15.00	0.37	0.37	0.00	0.23	0.20
CZ	Changjiangsanjiaozhou	50 m	13.00	0.00	0.00	0.00	0.00	0.00
HN	Hainan	120 m	8.00	0.00	0.00	0.00	0.13	0.00
GF	Shangrao, Jiangxi	500 m	15.00	0.06	0.06	0.00	0.00	0.00
HS	Inner Mongolia	1000 m	8.00	0.00	0.00	0.69	0.00	0.00
IM	Earlangshan, Inner Mongolia	1500 m	13.00	0.65	0.73	0.23	0.58	0.62
NJ	Aksu, Xinjiang	1700 m	14.00	0.23	0.23	0.69	0.19	0.18
DL	Qinhai, haixizhou, Dula	3000 m	8.00	0.56	0.56	0.69	0.56	0.56
BG	Bange, Tibet	4000 m	17.00	0.67	0.67	0.67	0.67	0.69
RT	Ritu, Tibet	4700 m	17.00	0.97	0.97	0.97	0.97	1.00


Genomic DNA was isolated from ear tissue using a modified commercial Kit protocol (Promega, the Wizard^®^). The quality and integrity of the purified DNA from each goat per sample location were determined using a Nanodrop 2000 (Thermo Fisher Scientific, DE) and pooled according to the concentration bases. The validations in the larger goat population were carried out by following sample data and locations. Highland group consisted of Ritu (RT, *n* = 17) and Bange (BG, *n* = 17) goats from the Ritu and Bange region of Tibet. The lowland group consisted of Dulan (DL, *n* = 8) and Nanjiang (NJ, *n* = 14) cashmere goats from Qinhai, Dulan, and Aksu areas of Xinjiang region, Inner Magnolia (IM, *n* = 13) from the Earlangshan region of Inner Mongolia, Hanshan (HS, *n* = 8) from Inner Magnolia city, Guangfeng (GF, *n* = 15) from Shangrao, Jiangxi., Hainan (HN, *n* = 8) from Hainan city, Changjiangsanjiaozhou (CZ, *n* = 13) from Changjiangsanjiaozhou city and Liaoning (LN, *n* = 15) cashmere goats from Gai Zhou, Liaoning, as shown in Table 1, Supplementary Table S1 and Supplementary Figure S1.

### Polymerase Chain Reaction (PCR) and Single-Nucleotide Polymorphisms (SNPs) detection

Sixteen pairs of primers were used for *DSG3* gene amplification and their sequences are shown in Table 2. Polymerase chain reaction (PCR) was optimized with a 50 ng DNA template using 2xTaq master mix (BioMed: Beijing Co., Ltd.), supplemented with ddH20 solutions and 10 pmol/l of forward and reverse primers and in a 30 ul mixture. The PCR was performed by initial denaturation for 5 min at 95°C, followed by 40 cycles at 95°C for 30 s, annealing at 60–63°C for 30 s and an extension at 72°C for 45 s, and a final extension at 72°C for 5 min and stored at 4°C. The PCR products were analyzed by running on 1.5% of gel electrophoresis that was made by a mixture of 0.75 g Regular Agarose Biowest (Gene Company) and 50 ml of 1x TAE buffer. The mixture was also added with 5 μl gold-view chemical for viewing the gel on UV transilluminator. The electrophoresis tank (Bio-rad Sub-Cell^®^ GT) and the PowerPac Basic (Bio-rad^®^) were used for running the electrophoresis. Five microliters of the PCR products were mixed with 10X loading dye, which is composed of 0.25% Bromophenol blue, 0.25% Xylene Cyanol FF, 15% Ficoll, and H2O. The mixture was loaded into each running well of 1.5% gel. The electrophoresis was run under 1X TAE buffer, carried out at a 75W constant, 147 V for 40 min. When electrophoresis was finalized, the gels were set on the UV transilluminator. All the purified PCR products were directly sequenced by the Sanger Sequence methods, using the ABI 3730 sequence analyzer (Applied Bio-System, United States) in both the directions, and using the forward primer. The sequences were assembled and analyzed for single-nucleotide polymorphism (SNPs), using the Seq Man program of Laser gene software. The SNPs were detected from Primer 4, Primer 5, Primer 6, Primer 8, Primer 9, Primer 11, Primer 14, and Primer 16 in the *DSG3* gene through DNA pooled strategies. Furthermore, these primers were used in large populations on an individual basis to identify polymorphism and the validation of the SNPs.

**Table 2:** Sequences of the primers.

PCR primer	5′ → 3′	PCR region	Anneal temp	Product length
Exon-1F DSG3	GGCCTCTTTTGCTTGTGAGAAA	25786301	61	1571
Exon-1R DSG3	CAGTCACGATCAAACGTTGAGTT	25784966		
Exon-2F DSG3	CAGTAGCCGGATTTTGGCAG	25788145	60	528
Exon-2R DSG3	ACTTGGCATGTGCTCTTGGA	25787897		
Exon-3F DSG3	ACCGATTCATTGAAACCCTGTT	25791050	61	412
Exon-3R DSG3	CTCCTGACCATCAAGGACCA	25790985		
Exon-4F DSG3	GGTCCTTGATGGTCAGGAGTC	25791491	64	331
Exon-4R DSG3	TCCAACAATCGCCCTGTCAA	25791351		
Exon-5F DSG3	AACATACACGACCTGCTCTGC	25794845	63	600
Exon-5R DSG3	AACCCCAACAGCCCTCATAA	25794593		
Exon-6F DSG3	TGCTCCCAAATGGACTCCC	25796339	63	365
Exon-6R DSG3	GCACAACTTGCTCTGGATAGTTC	25796111		
Exon-7F DSG3	GCAGTGATAAAGGTAATGTGTTAT	25798279	60	422
Exon-7R DSG3	GCAGGAAATTTAAAACCCAGGCT	25798137		
Exon-8F DSG3	ATGAGGCAACTTCGCCAGTG	25799330	60	337
Exon-8R DSG3	GAGTTCTCTGGTCCTCTTACCTG	25799057		
Exon-9F DSG3	TGTGCTTAAAACAGGCACACT	25802312	59	599
Exon-9R DSG3	AGGGACCAGCAAAGAGACAAT	25802125		
Exon-10FDSG3	GCAAGCCAGCAAATCCTGAA	25802738	60	415
Exon-10RDSG3	CCCCACAGGCTTTGTCTACG	25802604		
Exon-11FDSG3	ACCCCTCTTCCTTCCCTCTTT	25803899	59	411
Exon-11RDSG3	GTGACACGTAGATTTTTGGCAC	25803733		
Exon-12FDSG3	TGGCAGTGGGTAAGTAGCTC	25804674	60	514
Exon-12RDSG3	GAAGGGGCAACTTGGACAAC	25804524		
Exon-13F DSG3	ATTCACGCACTGATATCCTTCT	25805338	59	324
Exon-13R DSG3	GACTTGAGAGTGTGGAGCAC	25805217		
Exon-14FDSG3	TGTTCTATGGACCCCTCAATCT	25807166	59	400
Exon-14RDSG3	TGCATGATAGTGTCAGGGAGAG	25807028		
Exon-15FDSG3	CACAAATACCGGTTTATCTGTTGA	25807700	51	366
Exon-15RDSG3	TGAACAGCAGAATGTAAAAATCAA	25807664		
Exon-16F DSG3	ATCTGGCTTGGTTTTCCATGTG	25817374	60	472
Exon-16R DSG3	GAGCAAAAGTGATGCAAGCGT	25817194		


### Statistical Analyses

Pairwise genetic distance (*F_ST_*) was calculated between High-Lowland and High-Midland and Mid-lowland goat populations to find out the genetic divergence as described by Weir and Cockerham (1984). Fisher’s exact test was estimated respectively, to observe a statistical significance. LD-block structure and parameter (D’ and *r*^2^) were figured using Haploview 4.2 software (Barrett et al., 2005). To explore the signature of selection and evolutionary change, the six SNPs (four Non-Synonymous and two Synonymous variants) were analyzed for the diversity of haplotype and their frequency was estimated using phase 2.1 (Stephens et al., 2001; Crawford et al., 2004). To obtain a reliable result, we used option – X 10 to increase final runs time and – C option for low- and high-land goat groups to ensure that any similarity to the haplotype is not taken into account. The haplotype was embedded in DNASP Version5 (Librado and Rozas, 2009) for network construction and phylogenetic diversity among the identified haplotypes was inferred through a median-joining network analysis using the method recommended by Bandelt et al. (1999). To detect the functions of three major non-synonymous SNPs in the *DSG3* gene, the SIFT software^1^ has been used. The protein sequence of Tibetan goats was compared with the reference protein sequence of goats desmoglein*-3*. The cutoff value in the SIFT program is a tolerance score of ≥ 0.05. The higher the tolerance score, the lower the expected functional impact of a particular amino acid substitution.

## Results

### Allele Frequency and Genetic Differentiation

**FIGURE 1:**
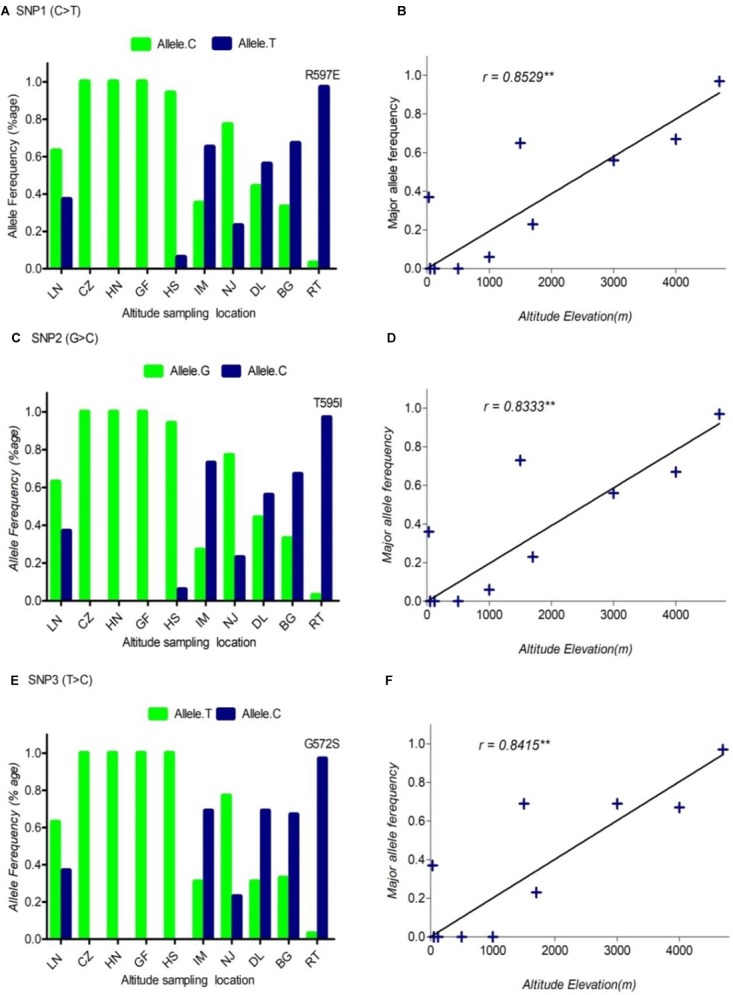
The pattern of allele frequency of SNP1 **(A)**, SNP2 **(C)** and SNP3 **(E)** of *DSG3* has continuously changed from sea level at 30 m (LN goat population) to the elevation at 4700 m (RT goat population). Blue, a frequency of the mutant allele, Green, a frequency of a reference allele in each goat population, respectively. The plot of the correlation analysis between major allele frequency of SNP1 **(B)**, SNP2 **(D)** and SNP3 **(F)** and sampling locations of 10 cashmere goat populations, *r* represents the correlation coefficient (*r* = 0.8529, *P* < 0.05). Asterisks (^∗∗^) represents very significantly.

**FIGURE 2:**
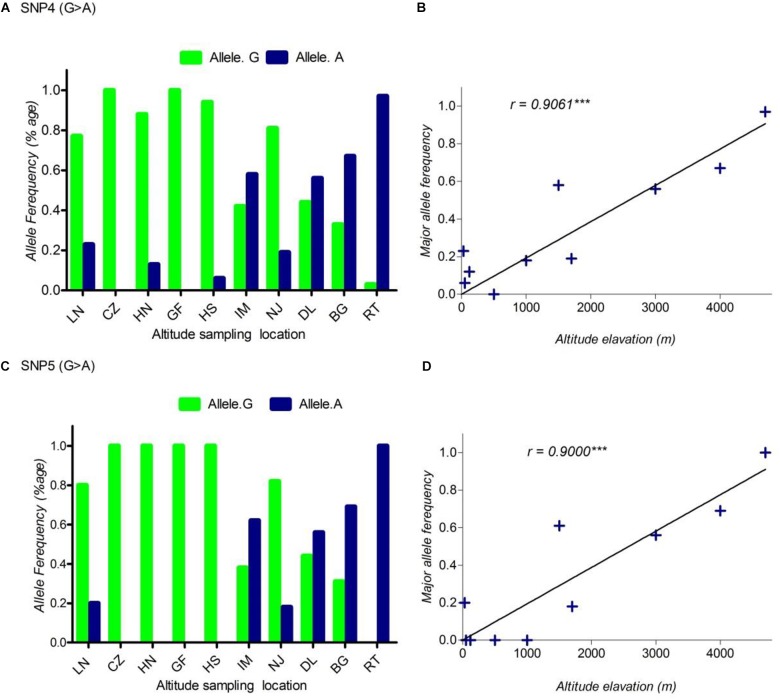
The pattern of allele frequency of SNP4 **(A)** and SNP5 **(C)** of *DSG3* has continuously changed from sea level at 30 m (LN goat population) to the elevation at 4700 m (RT goat population). Blue, a frequency of the mutant allele, Green, a frequency of a reference allele in each goat population, respectively. The plot of the correlation analysis between major allele frequency of SNP4 **(B)**, SNP5 **(D)** and sampling locations of 10 cashmere goat populations, *r* represents the correlation coefficient (*r* = 0.9061, *P* < 0.05). Asterisks (^∗∗∗^) represents extremely significantly.

In this study, we sampled 128 Chinese indigenous goats belonging to ten populations living across a wide distribution of altitudes. The ten populations ranged from sea level (30 m) to high altitude (4700 m) (Table 1 and Supplementary Table S1). To find out the polymorphism and to measure allele frequencies of the *DSG3* gene accurately, we resequenced the entire genomic region of the *DSG3* gene. Among the 27 SNPs that were identified in *DSG3*, five SNPs showed the most significant differentiation in the analysis of allele frequency and global *F_ST_*, including three non-synonymous SNPs, SNP1 (R597E, Chr24: 25794694, exon 5), SNP2 (T595I, Chr24: 25794695, exon 5), and SNP3 (G572S, Chr24: 25794771, exon 5), and two synonymous SNPs, SNP4 (Chr24: 25799255, exon 8), and SNP5 (Chr24: 25817330, exon 16) (Table 3). A striking change in allele frequency with elevation in altitude was observed in goat population. For example, low R597E allele frequencies in *DSG3* were found in lowland populations including CZ (0.00, 50m), HN (0.00, 120 m), and HS (0.00, 1000 m), but were elevated in the IM (0.65, 1500 m), DL (0.56, 3000 m), BG (0.67, 4000 m), and reached the highest frequency in the RT (0.97, 4700 m) population (Table 1, Supplementary Table S2, and Figure 1A). The other four SNPs in *DSG3* exhibited a similar pattern of change (Table 1, Supplementary Table S2, and Figures 1, 2). All these results indicated that the frequencies of the five variants in the *DSG3* locus remain rare in the lowland goat population but increased with the elevation of altitude.

**Table 3:** Correlation and global *F_ST_* value of five mutation loci in DSG3 gene.

SNPs	Genomic location	Reference allele	Mutation allele	Pearson r	*G. F_ST_* values
SNP1 (R597E)	Chr24: 25794694	C	T	0.8529	0.50
SNP2 (T595I)	Chr24: 25794695	G	C	0.8333	0.55
SNP3 (G572S)	Chr24: 25794771	T	C	0.8415	0.50
SNP4	Chr24: 25799255	G	A	0.9061	0.47
SNP5	Chr24: 25817330	G	A	0.9000	0.61


Using allele frequencies of the three non-synonymous and two synonymous SNPs measured above, we measured the correlation co-efficiency of variant allele frequency per population versus the altitude of sampling location using Graph Pad Prism 5 software. The result of this analysis showed a significant positive linear correlation between the elevated altitude and the frequency of a variant allele in *DSG3* (Table 3 and Supplementary Table S3). The linear correlation coefficient between the elevated altitude and the frequencies of variant allele was *r* = 0.85, *P* < 0.05 for SNP1 (Figure 1B), *r* = 0.83, *P* < 0.05 for SNP2 (Figure 1D), *r* = 0.84, *P* < 0.05 for SNP3 (Figure 1F). The linear correlation coefficient was *r* = 0.91, *P* < 0.05 for SNP 4 (Figure 2B), and *r* = 0.90, *P* < 0.05 for SNP5 (Table 3, Supplementary Table S3, and Figure 2D). The significant positive correlation between the elevated altitudes and the variant allele frequency at the *DSG3* locus in ten Chinese native goat populations strongly suggested that the candidate gene *DSG3* potentially contributed to the high-altitude adaptation in goats.

To further understand the genetic differentiation that occurred between the lowland and highland goat populations, we first estimated the global *F_ST_* value for all the identified loci. The mean *F_ST_* value was 0.24, indicating the high genetic divergence in these ten Chinese native goat populations, which is mainly attributed to the divergence between the lowland and highland goat populations. The global *F_ST_* of all the 27 loci ranged from 0.0 to 0.61 (Supplementary Table S2). The three most divergent non-synonymous SNPs had the value that ranged from 0.51 (SNP1) and 0.52 (SNP2) to 0.55 (SNP3), whereas the top two synonymous SNPs had a global *F_ST_* of 0.47 (SNP4) and 0.61 (SNP5) (Table 3 and Supplementary Table S2). Subsequently, the pairwise genetic distance (*F_ST_*) was measured between High-Low, High-Mid, and Mid-Low altitude goat populations (Table 4). The *F_ST_* values of non-synonymous variants between high-altitude and low-altitude populations were 0.42 (SNP2, *P* < 0.001) and 0.45 (SNP1/SNP3, *P* < 0.001), which reflected a remarkable genetic differentiation (Table 4). Similarly, the *F_ST_* values of the two synonymous substitutes were 0.47 (SNP4, *P* < 0.001) and 0.58 (SNP5, *P* < 0.001). These results showed high genetic differentiation between High-Low altitude goat populations compared with the High-Mid and Mid-Low altitude goat populations (Table 4). Taken together, both of global *F_ST_* values and pairwise genetic distance (*F_ST_)* values demonstrate a remarkable population differentiation between the high- and low-altitude goat populations.

**Table 4:** Pairwise genetic distances (*F_ST_*) & Fisher exact test (*p*-value) calculated between highland and reference goat populations.

Chromosome position	SNPs	M allele	Anno.	H_M	*P*-value	H_L	*P*-value	M_L	*P*-value

				(*F_ST_*)		(*F_ST_*)		(*F_ST_*)	
Chr24:25791412		T		0.06	0.00	0.08	1.13E-04	0.00	0.88
Chr24:25794517		G		0.05	0.00	0.12	1.30E-07	0.02	0.03
Chr24:25794535		A		0.05	0.00	0.01	0.12	0.03	0.13
Chr24:25794564		A		0.03	0.06	0.00	1.00	0.03	0.06
Chr24:25794694	SNP1^∗^	T	R597E	0.25	3.03E-12	0.45	1.22E-20	0.04	0.02
Chr24:25794695	SNP2^∗^	C	T595I	0.25	3.03E-12	0.42	2.17E-19	0.03	0.04
Chr24:25794700		A		0.03	0.06	0.00	0.25	0.03	0.68
Chr24:25794771	SNP3^∗^	C	G572S	0.20	3.24E-10	0.45	5.25E-20	0.06	0.00
Chr24:25794881		A		0.10	1.40E-05	0.18	3.53E-09	0.01	0.13
Chr24:25794882		A		0.00	1.00	0.00	1.00	0.00	1.00
Chr24:25794934		A		0.00	1.00	0.12	1.30E-06	0.11	1.30E-06
Chr24:25796278		C		0.10	0.00	0.19	2.75E-09	0.02	0.04
Chr24:25796283		C		0.04	0.02	0.06	0.00	0.00	0.41
Chr24:25799144		G		0.03	0.06	0.00	1.00	0.03	0.21
Chr24:25799255	SNP4^∗^	G		0.25	1.73E-13	0.47	2.08E-22	0.05	0.01
Chr24:25799281		G		0.01	1.00	0.03	0.01	0.06	0.01
Chr24:25801885		C		0.01	0.06	0.11	5.71E-08	0.07	0.00
Chr24:25802080		T		0.01	1.00	0.03	0.03	0.06	0.03
Chr24:25802106		G		0.00	1.00	0.01	0.50	0.01	0.50
Chr24:25803941		C		0.08	0.01	0.27	1.01E-10	0.09	0.00
Chr24:25803953		G		0.01	0.50	0.00	1.00	0.00	1.00
Chr24:25807002		T		0.00	2.19E-13	0.00	7.22E-17	0.00	0.35
Chr24:25807209		T		0.06	0.01	0.14	6.14E-07	0.02	0.02
Chr24:25807240		T		0.00	1.00	0.09	7.26E-06	0.09	7.27E-06
Chr24:25817330	SNP5^∗^	A		0.31	8.94E-16	0.58	4.25E-26	0.07	0.01
Chr24:25817361		G		0.00	1.00	0.00	1.00	0.00	0.50
Chr24:25817366		G		0.03	0.06	0.02	0.10	0.00	0.80


*Top five SNPs are shown with asterisk.*

### Linkage Disequilibrium (LD) and Haplotype Network Analysis

**FIGURE 3:**
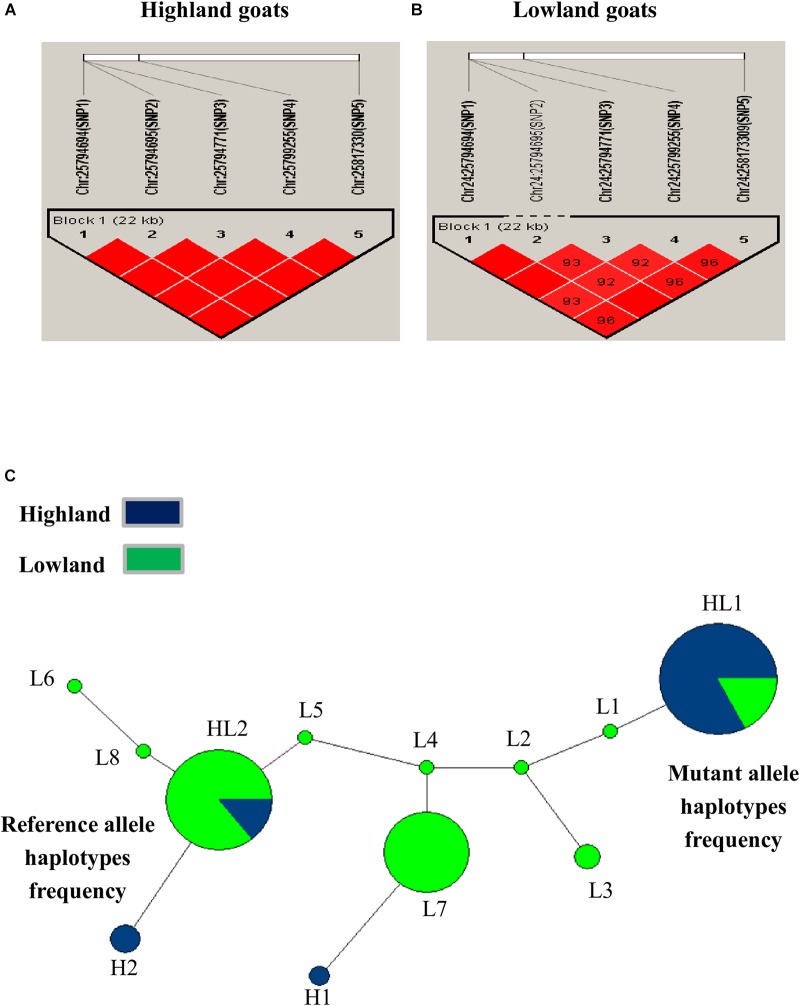
**(A)** Linkage disequilibrium of non-synonymous variants (SNP1, SNP2, and SNP3) and synonymous substitutions (SNP4 and SNP5) in highland goat populations; RT and BG. **(B)** Linkage disequilibrium of non-synonymous variants (SNP1, SNP2, and SNP3) and synonymous substitutions (SNP4 and SNP5) in lowland goat populations; LN, CZ, HN, GF, HS, IM, NJ, and DL. Strengths of the LD between two SNPs were indicated by the color scheme (a bright red diamond indicates a strong correlation) by the pairwise D° values given as the percentage in each diamond (a bright red diamond indicates D° = 1.00). **(C)** Haplotype networks of non-synonymous variants (SNP1, SNP2, and SNP3, T480N) and synonymous substitutions (SNP4 and SNP5) among lowland and highland goat populations. Each node represents a haplotype, with the size of the circle proportional to frequency. The length of a branch is proportional to the number of nucleotide differences. Circles are color code according to the population. Blue: highland (RT and BG), Green: lowland (LN, CZ, HN, GF, HS, IM, NJ, and DL), abbreviation: HL, high and low altitude; H, high altitude; L, low altitude.

The LD haploblock pattern was used to analyze the *DSG3* loci (Supplementary Figure S2). We constructed LD blocks using the five significant SNPs and calculated D°, *r*^2^, and an algorithm of the odds (LOD). The variant allele of these five SNPs was tightly linked as a Haplo-Block on chromosome 24 in highland goat populations (RT-BG), with the three non-synonymous SNPs in the main position and showed strong correlation by D°= 1.0 -1.0, *r*^2^ = 0.63–1.00, and LOD = 5.19–9.82 (Table 5 and Figure 3A). In contrast, the linkage of three non-synonymous SNPs and two synonymous SNPs was loosely found in lowland goat populations (DL-NJ-AB-LN-GF-CZ-HN-HS). However, the LD level of SNPs varied from D = 0.58–1, *r*^2^ = 0.10–0.94, and LOD = 2.85–31.71 (Table 5 and Figure 3B). The haplotype pattern was analyzed to detect the effect of natural selection on selected genes, including *DSG3*. Twelve haplotypes were obtained from four non-synonymous (SNP1/R392Q, SNP2/T595I, SNP3/G572S, and T480N) and two from synonymous SNPs (SNP4 and SNP5) from 128 goats from lowland and highland goat populations. The top three haplotypes were detected: HL1, HL2, and L7 with a frequency of 42%, 40%, and 30%, respectively (Figure 3C). Among these haplotypes, HL1 showed a remarkably higher haplotype frequency of a mutant allele (TCCCAA) in highland population than in lowland populations (18.0%). However, HL2 indicated a much lower haplotype frequency of the reference allele (CGTAGG) in the highland (7.0%) than in the lowland population (42%). L7 showed a haplotype frequency of (CGTCGG) in lowland (31%) and indicated a separate haplotype cluster in lowland populations. These results suggested that the haplotype TCCCAA of the *DSG3* gene has been selected during the high-altitude adaptation of the Tibetan goat.

**Table 5:** Pairwise linkage disequilibrium of the non-synonymous and synonymous SNPs of DSG3 in the highland and lowland goat populations.

SNPS		Highland goat	Lowland goat	Highland goat	Lowland goat	Highland goat	Lowland goat

SNP1	SNP2	D’		*r*^2^		LOD	
SNP1 (C > T)	SNP2 (G > C)	1.00	1.00	1.00	0.94	9.82	31.71
SNP1 (C > T)	SNP3 (T > C)	1.00	1.00	1.00	0.91	9.82	30.46
SNP1 (C > T)	SNP4 (G > A)	1.00	0.93	1.00	0.79	9.82	22.95
SNP1 (C > T)	SNP5 (G > A)	1.00	0.96	0.63	0.73	5.19	21.95
SNP2 (G > C)	SNP3 (T > C)	1.00	0.94	1.00	0.86	9.82	27.56
SNP2 (G > C)	SNP4 (G > A)	1.00	0.93	1.00	0.74	9.82	21.87
SNP2 (G > C)	SNP5 (G > A)	1.00	1.00	0.63	0.75	5.19	24.01
SNP3 (T > C)	SNP4 (G > A)	1.00	0.93	1.00	0.71	9.82	21.12
SNP3 (T > C)	SNP5 (G > A)	1.00	0.96	0.63	0.67	5.19	20.58
SNP4 (G > A)	SNP5 (G > A)	1.00	0.96	0.63	0.84	5.19	25.26


### Prediction of Three Non-synonymous SNPs

An online tool SIFT was used to investigate the potential impact of the non-synonymous substitutions on protein structure and function. SIFT aligns a query sequence and a subject amino acid sequence to determine the effect of an amino acid substitution. SIFT software takes protein FASTA sequences as input and is aligned with PSI-BLAST. In our result analysis, we found three nsSNPs to be predicated, as shown in Table 6, by using SIFT software.

**Table 6:** Predications of non-synonymous SNPs function by SIFT software.

nsSNPs	Positions	Reference allele	Mutant allele	Amino acid changed	Predications	Score
SNP1	597	C	T	R597E	Affect protein function	0.000
SNP2	591	G	C	T595I	Affect protein function	0.000
SNP3	572	T	C	G572S	Affect protein function	0.000


*Amino acid substitutions changed in Tibetan goat protein sequences may have been predicted to modify the function of *DSG3* gene (low certainty). The cutoff value in the SIFT program is a tolerance score of ≥ 0.05. The higher the tolerance score, the less functional impact a particular amino acid substitution is expected to have.*

## Discussion

There are many animal species that live on the Tibetan plateau. The plateau consists of 25% of Mainland China, with an average altitude exceeding 4,500 m (Thompson et al., 2000; Wei et al., 2016). Up to now, genomic studies indicated several significant pathways and functional categories including energy metabolism and oxygen transmission, DNA repair, and ATPase production, in response to hypoxia at the Tibetan plateau (Qiu et al., 2012; Ge et al., 2013; Li et al., 2013; Qu et al., 2013; Xiang et al., 2013). Moreover, SNP data have been obtained from population surveys to find the mechanism underlying plateau adaptation (Wei et al., 2016) and have successfully identified candidate genes *EGLN1, PPARA, and EPAS1* as transcription factors with a pivotal role in adaptation (Buroker et al., 2012; Xiang et al., 2013; Gou et al., 2014; Wang et al., 2014).

In our previous exome sequencing of 330 cashmere goats, we identified a genomic region containing a desmosome gene *DSG3* (Desomoglein3) under strong selection. *DSG3* gene is a desomoglein protein-coding gene located in a cluster on goat chromosome 24, consisting of 16 exons.

In this study, the entire region of (44 kbp) *DSG3* was sequenced. Subsequently, the single-nucleotide polymorphism was identified in a large population of indigenous Chinese goats to determine the genetic difference between the lowland and highland goat populations. Our results showed polymorphism in *DSG3* (a) segregated indigenous Chinese goat population in two clusters; (b) a significant positive linear correlation between the elevated altitude and the frequency of a mutant allele; (c) non-synonymous candidate mutations indicating the contribution of gene in high-altitude adaptation of Tibetan goats.

Moreover, our previous studies suggested that the positive directional selections of genes such as *EPAS1* (Endothelial Per-ARNT-Sim (PAS) domain protein 1), *EDNRA* (Endothelin-1 receptor Precursor), *SIRT1* (Sirtuin type 1), and ryanodine receptor *1 (RYR1)* are linked to hypoxia. *EPAS1, RYR1, DSG2* (Desomgelin2) are related to cardiomyopathy and *PTPRJ* (R*eceptor-type tyrosine-protein phosphatase eta), FUT1* (fucosyltransferase1), *HEG1* (heart development protein with EGF-like domains 1), *PTPRZ1* (tyrosine phosphatase receptor-type Z polypeptide 1), *SIGLEC1* (Sialic acid-binding Ig-like lectin 1 sialoadhesin), *NPC1L1* (Niemann-Pick disease-type C1 gene-like 1), and *NES* (Nestin) are connected with the cardiovascular system, whereas *DSG3* is important in maintaining the normal structure and function of hairs (Song et al., 2016). Also, some studies suggested that the *DSG3* gene is associated with skin development (Koch et al., 1998; Song et al., 2016).

A high phenotypic variation exists in the Cashmere goat introduced by natural and artificial selection for over 10,000 years, in line with human demand (Naderi et al., 2008; Wang et al., 2016). Owing to evolutionary and artificial selection, the goat has been adapting to different harsh environmental conditions (Song et al., 2016). Multiple genes are described in the significant role of hair and fleece growth in cashmere goats, such as the *POU1F1* and *PRL* genes associated with cashmere yield, diameter, and length, whereas the *LHX2 and LIM* genes regulate the generation of hair. The *FGF5* gene is a key regulator that controls hair length and the *FGF9* gene to promote the regeneration of hair follicle after wounding. The *WNT2* gene is involved in the initiation of hair follicle (Wang et al., 2016). Furthermore, the *DSG1* gene is involved in the communication of hair follicle cell and morphogenesis of hair follicle (Hanakawa et al., 2004; Dong et al., 2013). The *DSG4* gene affects wool traits and is responsible for coat color in Cashmere goats (E et al., 2016). Furthermore, the *DSG3* gene may have linked with additional fiber growth in the cashmere goats. Other studies have reported that the *DSG1, DSG3*, and *DGS4* genes were expressed in keratinocytes of the basal and immediate suprabasal cell layers (Koch et al., 1998; Bazzi et al., 2006; Dusek et al., 2006). According to Nazari-Ghadikolaei et al. (2018), keratinocytes produce keratin, the main protein for hair, nail, and skin synthesis (Nazari-Ghadikolaei et al., 2018).

The biological function of *DSG3* is keratinocyte cell-to-cell adhesion in the basal and suprabasal layers of stratified squamous epithelia. *DSG3* has been shown to be the self-antigen for autoantibodies in the pemphigus vulgaris (PV) disease and is known as PV antigen (PVA) (Koch et al., 1997). In past studies of mice and humans, *DSG3* was predominantly expressed in the outer layer of skin tissues such as basal and suprabasal cell layers of epidermis (Kljuic et al., 2003), whereas other members of the desmoglein gene family *DSG1* are only expressed in suprabasal cell layers and those of *DSG2* in the basal cell layer (Koch et al., 1997). *DSG3* provide desmogleing compensations with *DSG1* in pemphigus foliaceus (PF), which is a potentially fatal autoimmune blistering skin disease in which autoantibodies against *DSG3* and *DSG1* cause loss of keratinocyte cell adhesion (Payne et al., 2005).

Similarly, desmoglein 3 (*DSG3*), a *SLC24A5* gene, is involved in lighter skin pigmentation in Europeans, showing strong signals of positive selection in the high-altitude populations (Huerta-Sanchez et al., 2013). Furthermore, desmoglein genes (*DSG1-4*) are expressed in the epidermis and myocardium tissue and their disruption causes some autoimmune diseases that affect the skin, heart, and mucous membranes in humans (Garrod and Chidgey, 2008; Thomason et al., 2010). Desmoglein3 (*DSG3*) has been identified as one of the autoantigens in an autoimmune blistering skin disease called pemphigus vulgaris (PV) (Stanley and Amagai, 2006; Hartlieb et al., 2013). In this disease, circulating autoantibodies targeting *DSG3* induce loss of cell cohesion within the epidermis and mucous membranes. *DSG3* was considered to be the new gene marker for terminal differentiation and was found to be a highly upregulated gene during early phase acute hypobaric hypoxia in the rats (Sharma et al., 2015). There were a few studies related to the functional role of *DSG3*; however, its clear function has not been established yet. It has been suggested that *DSG3* may involve more than cell-cell adhesion (Tsang et al., 2010). The expression of the *DSG3* gene mostly occurred in the stratified epithelial tissues (Stanley and Amagai, 2006). An epithelial tissue plays an essential role in the diseases of altitude because these tissue cells are the lining of the alveoli of lungs (air sacs) and the blood vessels (endothelium). These tissues have no blood vessels and thus must receive oxygen from the blood vessels in the adjacent connective tissue. For this reason, the tissues are particularly susceptible to damage from low oxygen at high altitude. High-altitude hypoxia impacts structures of vital cells such as sodium and potassium pumps and transcription of the genes slows the sodium and potassium pumps in the alveoli of the lungs. Decreasing the number of these pumps contributes to the accumulation of water in the alveoli and causes high-altitude pulmonary edema (Litch, 2006). Several candidate genes have a key role in controlling the complex cellular processes, energy metabolism, oxygen respiration chain, and reform adenosine triphosphate (ATP) in the cell tissues (Niu et al., 2017). During the evolutionary process, the genes functioning within the oxygen respirations chain have been considered under positive selections. These genes are important for adaptation to high-altitude environments (Xu et al., 2007). Our current study has also shown the importance of Desmosomes genes in high-altitude hypoxia adaptation which has been supported by the SNP data validation analysis.

To our knowledge, there are no research publications related to polymorphism of the *DSG3* gene and its role for adaptation to hypoxia at high altitude. In this study, twenty-seven SNPs sites were found from the whole sequence of the *DSG3* gene. The allele frequency of variants was observed to be very low in lowland than in highland goat populations. These results have indicated that allele frequencies of variants might be associated with evolution, selection, genetic variation, and ecological conditions (Li et al., 2004a; Wang et al., 2011). Pearson correlation analysis of SNP1, SNP2, SNP3, SNP4, and SNP5 showed a significant relationship between allele frequency and elevation of altitude. These results are in line with the *EPAS1* gene in the Cashmere goat and Tibetan dog (Fan et al., 2015; Song et al., 2016).

Similarly, the global *F_ST_* value varied among the Chinese indigenous goat population at all the SNPs sites. The pairwise genetic distances were an ideal index to measure the polymorphism between the groups. Some exons and introns were excluded from further analysis due to a lower pairwise *F_ST_* value. Three non-synonymous and two synonymous mutations have shown large polymorphisms and pairwise genetic distance in the *DGS3* gene. Furthermore, this segregated the goat population in distinct branches, as discussed in previously reported studies (Xiang-Long and Valentini, 2004; Di et al., 2011; Ling et al., 2012). Linkage disequilibrium and haplotype analysis showed that the non-synonymous SNPs are highly linked with synonymous SNPs and had higher haplotype frequency variations in the genome region of *DSG3* in highland goat populations (Nordborg and Tavaré, 2002; Conrad et al., 2006; Li et al., 2006).

**FIGURE 4:**
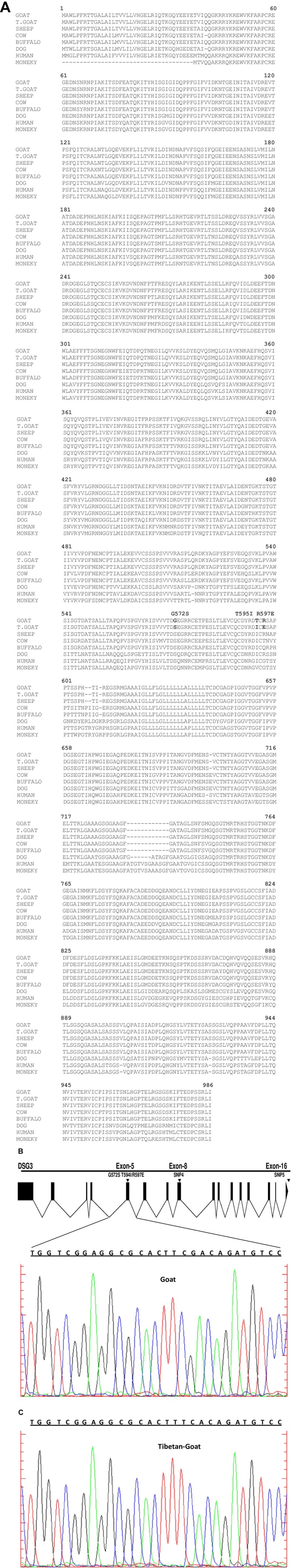
**(A)** Protein sequence alignment among different mammalian aligned by cluster omega (Sievers et al., 2011) predicated non-synonymous mutation in Tibetan goat, a conserver site or polymorphism. The dashes indicate deletions. **(B)** Sanger sequence of blue and black region showed nucleotide position changed in lowland goat, blue indicated nucleotide C, while black indicated nucleotide G. **(C)** Sanger sequence of red and blue region indicated nucleotide position changed in Tibetan goat, red indicated nucleotide T, while Blue indicated nucleotide C.

In this study, three non-synonymous mutations are found from the *DSG3* gene, out of which one mutation G597S is similar to the mutation G305S at *EPAS1* and G14S at *HBB* (Gou et al., 2014; Fan et al., 2015) in the dog. The G305S is an important mutation with a protein functional change in the PAS domain and has a major effect on the blood flow resistance (Adzhubei et al., 2010; Gou et al., 2014). Moreover, it has been demonstrated as a strongly conserved amino acid site at *EPAS1* in the dog (Fan et al., 2015). Therefore, the non-synonymous mutation G597S is likely to affect the function of the *DSG3* genes (Figures 4A–C) and suggests a role in high-altitude hypoxic adaptation.

Gene mutations and polymorphism are important to understand selection, adaptation, and biological evolutionary processes and to identify the genes related to genetic disease and particular traits (Akey et al., 2002; Nielsen, 2005). The single-nucleotide polymorphism (SNPs) variations in the exonic region of DNA sequences have shown many evolutionary changes. Although the substitution of non-synonymous mutations changed the coding sequence and altered the function of the protein, whereas the synonymous SNP did not change the coding sequence, it may affect the timing of cotranslational protein folding (Sun et al., 2013). In our studies, we have explored five significant SNPs in the most important skin gene *DSG3*, including three non-synonymous and two synonymous substitutes. They have shown a significant positive linear correlation between mutant allele frequency and elevated altitude in the Chinese indigenous goat population.

Future investigations of the *DSG3* gene in goats should focus on the expression level of the gene associated with mutations and altitude. This study has provided the information on the potential function of an allele of *DSG3* in high adaptation and strengthens the understanding of adaptation of the Tibetan goat to the extremely harsh environment of high altitude.

## Conclusion

Our research has, for the first time, shown the role of the Desmosomes gene for the high-altitude hypoxia adaptation and abundant genetic diversity between goat populations. We found that three non-synonymous candidate mutations (R597E, T595I, and G572S) and two synonymous substitutions significantly segregated these goat populations. Moreover, these results indicated the contribution of *DSG3* in high-altitude adaptation and provided new insights for Tibetan cashmere goats.

## Author Contributions

CK and LJ wrote the manuscript. XH, QZ, and YP contributed to sample collections and designing the manuscripts. CK and SS has performed the data analysis. YM and LJ conceived the study design. YM, KKM, and AAK interpreted the results and revised the manuscript. All the authors read and approved the final manuscript.

## Conflict of Interest Statement

The authors declare that the research was conducted in the absence of any commercial or financial relationships that could be construed as a potential conflict of interest.

## Footnotes

1 http://sift.bii.a-star.edu.sg/
