# Supplementary material for: Sequence Characterization of DSG3 Gene to Know Its Role in High-Altitude Hypoxia Adaptation in the Chinese Cashmere Goat
Source: Front Genet. 2018 Nov 19;9:553. doi: 10.3389/fgene.2018.00553 (PMC6254015; doi:10.3389/fgene.2018.00553)
Supplement: Supplementary file 1 [file Data_Sheet_1.PDF]

Supplementary Material

Sequence characterization of *DSG3* gene to know its role in high altitude hypoxia adaptation in the Chinese cashmere goats

Chandar Kumar*, Ma Yue Hui

*** Correspondence:** yuehui.ma@263.net;

1 Supplementary Figures

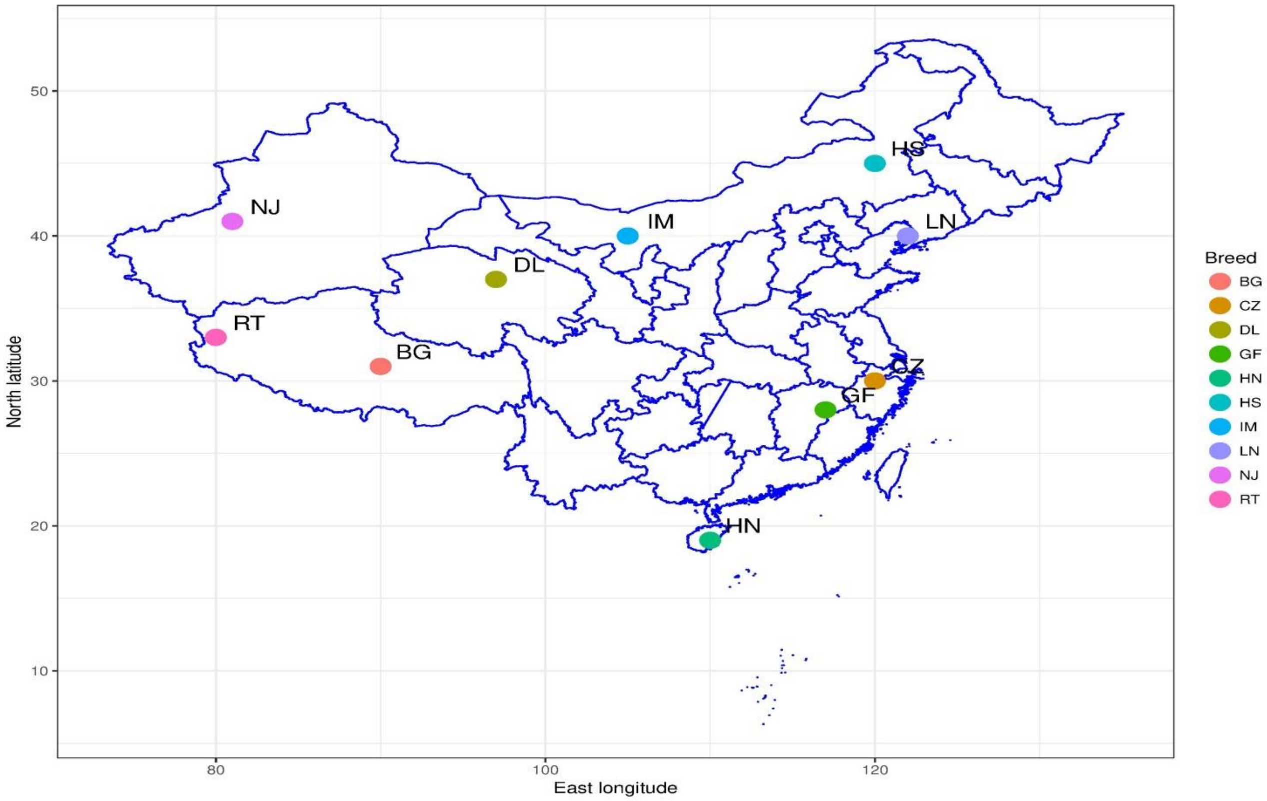

Fig S1. Geographical locations of ten Chinese cashmere goat populations

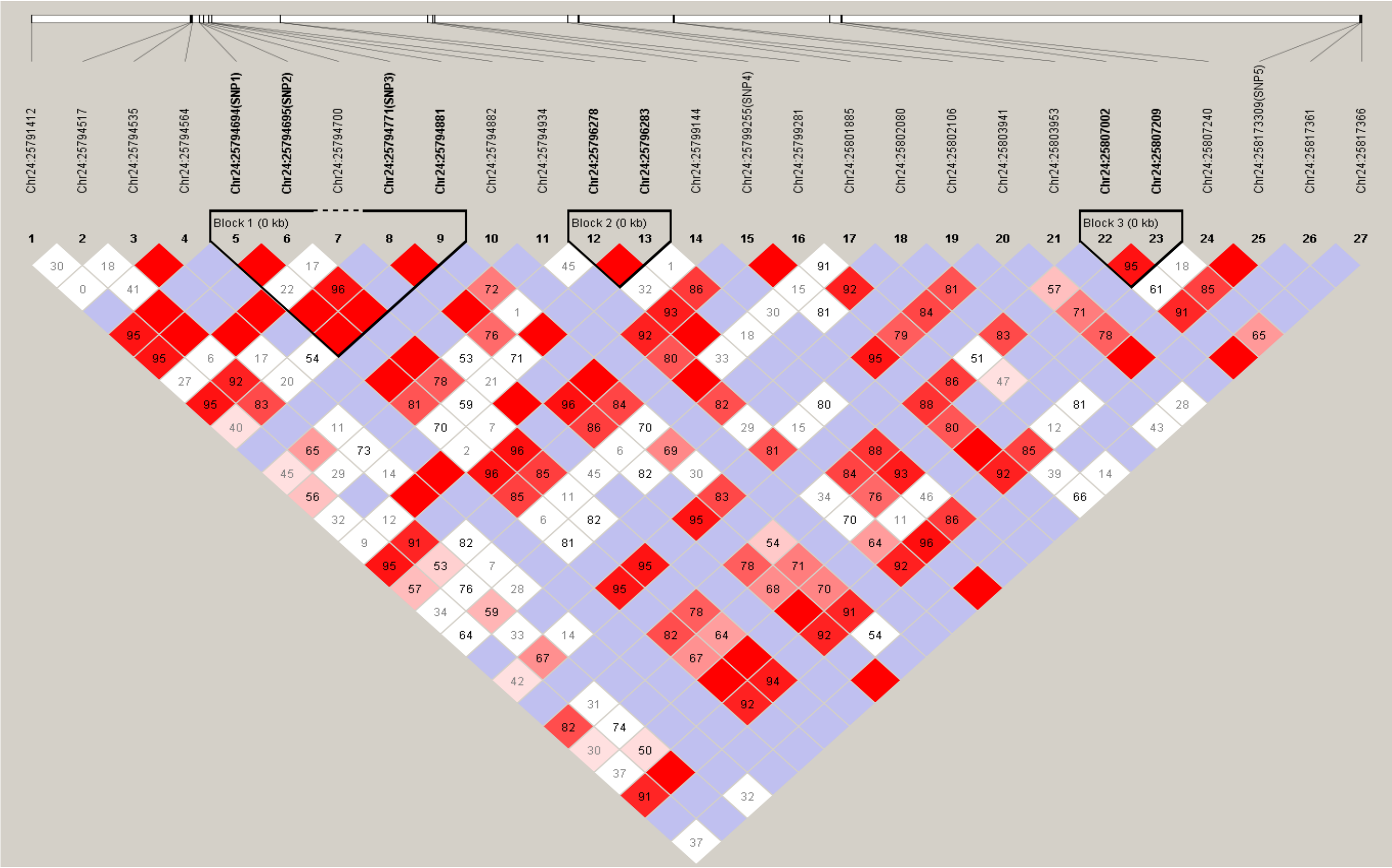

Fig S2. Linkage disequilibrium plot of 27 SNP loci of *DSG3*

frontiers
