# Supplementary material for: Sequence Characterization of DSG3 Gene to Know Its Role in High-Altitude Hypoxia Adaptation in the Chinese Cashmere Goat
Source: Front Genet. 2018 Nov 19;9:553. doi: 10.3389/fgene.2018.00553 (PMC6254015; doi:10.3389/fgene.2018.00553)
Supplement: Supplementary file 2 [file Data_Sheet_2.PDF]

Supplementary Material

1 Supplementary Table

Table S1. Sampling locations and size of indigenous Chinese goat populations

**Cashmere goat**	**Sample Location**	**Sample Size Total: 128**	**Altitude (m)**
**Low Altitude**			
Liaoning (LN)	Gai Zhou, Liaoning	15	30 m
Changjiangsanjiaozhou (CZ)	Changjiangsanjiaozhou	13	50 m
Hainan goat (HN)	Hainan	08	120 m
Guangfeng (GF)	Shangrao, Jiangxi.	15	500 m
Hanshan (HS)	Inner Mongolia	08	1000 m
Inner Magnolia (IM)	Earlangshan, Inner Mongolia	13	1500 m
**Middle Altitude**			
Nanjiang (NJ)	Aksu, Xinjiang	14	1700 m
Dulan (DL)	Qinhai, haixizhou, Dulan	08	3000 m
**High Altitude**			
Bange (BG)	Bange, Tibet	17	4000 m
Ritu (RT)	Ritu, Tibet	17	4700 m

TableS2. Allele frequencies and global FST values distribution in lowland and highland goat populations

Chromosome Position	SNPs	Mutant allele	ANNO.	Lowland								Highland		G.FST
				LN	CZ	HN	GF	HS	IM	NJ	DL	BG	RT	
Chr24:25791412		T		0.71	0.67	0.50	0.53	0.62	0.92	0.50	1.00	0.75	1.00	0.19
Chr24:25794517		G		1.00	0.61	0.81	0.50	0.87	1.00	0.93	0.87	1.00	1.00	0.24
Chr24:25794535		A		1.00	1.00	1.00	0.94	1.00	0.88	0.96	0.81	1.00	1.00	0.11
Chr24:25794564		A		1.00	1.00	1.00	1.00	1.00	1.00	1.00	0.87	1.00	1.00	0.11
Chr24:25794694	SNP1	T	R597E	0.37	0.00	0.00	0.00	0.06	0.65	0.23	0.56	0.67	0.97	0.50
Chr24:25794695	SNP2	C	T595I	0.36	0.00	0.00	0.00	0.06	0.73	0.23	0.56	0.67	0.97	0.55
Chr24:25794700		A		1.00	1.00	1.00	1.00	1.00	0.88	1.00	0.87	1.00	1.00	0.11
Chr24:25794771	SNP3	C	G572S	0.37	0.00	0.00	0.00	0.00	0.69	0.23	0.69	0.67	0.97	0.52
Chr24:25794881		A		0.87	0.50	0.44	0.25	0.62	0.96	0.61	0.87	0.92	0.97	0.29
Chr24:25794882		A		1.00	1.00	1.00	1.00	0.93	1.00	1.00	1.00	1.00	1.00	0.00
Chr24:25794934		A		0.93	0.50	0.69	0.50	0.94	1.00	0.96	1.00	0.96	1.00	0.30
Chr24:25796278		C		0.30	0.65	0.50	0.50	0.18	0.69	0.61	0.69	0.71	1.00	0.25
Chr24:25796283		C		0.67	0.96	1.00	0.84	0.87	0.81	0.82	1.00	0.96	1.00	0.12

Chr24:25799144		G		1.00	1.00	1.00	1.00	1.00	0.92	1.00	0.87	1.00	1.00	0.11
Chr24:25799255	SNP4	A		0.23	0.00	0.12	0.00	0.06	0.58	0.19	0.56	0.67	0.97	0.47
Chr24:25799281		G		0.90	1.00	0.81	0.66	0.93	0.96	0.96	1.00	0.96	1.00	0.15
Chr24:25801885		C		0.67	1.00	1.00	1.00	0.33	0.50	0.93	1.00	1.00	1.00	0.44
Chr24:25802080		T		0.93	1.00	0.78	0.71	1.00	1.00	0.96	1.00	0.96	1.00	0.17
Chr24:25802106		G		1.00	1.00	0.86	1.00	1.00	1.00	1.00	1.00	1.00	1.00	0.13
Chr24:25803941		C		0.83	0.46	0.44	0.25	0.69	1.00	0.82	0.94	0.96	0.97	0.35
Chr24:25803953		G		1.00	1.00	0.94	1.00	1.00	1.00	0.96	1.00	1.00	1.00	0.05
Chr24:25807002		T		0.13	0.54	0.14	0.09	0.06	0.75	0.18	0.56	0.69	0.94	0.00
Chr24:25807209		T		0.27	0.69	0.64	0.78	0.44	0.83	0.64	0.94	0.77	1.00	0.22
Chr24:25807240		T		1.00	0.38	0.86	0.91	0.94	1.00	1.00	1.00	1.00	1.00	0.42
Chr24:25817330	SNP5	A		0.20	0.00	0.00	0.00	0.00	0.61	0.18	0.56	0.69	1.00	0.61
Chr24:25817361		G		1.00	1.00	1.00	1.00	1.00	0.92	1.00	1.00	1.00	1.00	0.08
Chr24:25817366		G		0.93	1.00	0.91	0.75	0.87	0.93	1.00	0.87	0.96	1.00	0.08

Table S3.Correlation analysis between the altitude of sampling locations and the mutant allele frequencies in the DSG3 gene

**SNP locus**	R^2	**Pearson *r***	***P*-value**
SNP1 (C>T)	0.7274	0.8529	0.0017**
SNP2 (G>C)	0.6943	0.8333	0.0028**
SNP3 (T>C)	0.6634	0.8145	0.0041**
SNP4 (G>A)	0.8209	0.9061	0.0003***
SNP5 (G>A)	0.8099	0.9000	0.0004***

represent a significant *P*-value of less than 0.05 ***
